# SURFIN_4.1_, a schizont-merozoite associated protein in the SURFIN family of *Plasmodium falciparum*

**DOI:** 10.1186/1475-2875-7-116

**Published:** 2008-07-01

**Authors:** Fingani A Mphande, Ulf Ribacke, Osamu Kaneko, Fred Kironde, Gerhard Winter, Mats Wahlgren

**Affiliations:** 1 Department of Microbiology, Tumor and Cell Biology (MTC) Karolinska Institutet and Swedish Institute for Infectious Disease Control, Nobel's väg 16, Box 280, SE-171 77, Stockholm, Sweden; 2 Department of Protozoology, Institute of Tropical Medicine, Nagasaki University, 1-12-4 Sakamoto, Nagasaki 852-8523, Japan; 3 Department of Biochemistry, Makerere University, Kampala, Uganda; 4 Department of General Practice, Flinders University, Adelaide, Australia

## Abstract

**Background:**

In its effort to survive the human immune system, *Plasmodium falciparum *uses several parasite-derived antigens most of which are expressed at the surface of the parasitized red blood cells (pRBCs). Recently SURFINs, a new family of antigens encoded by the *surf *multi-gene family, has been reported. One member of the family, SURFIN_4.2_, was found present both at the pRBC-surface and at the merozoite apex.

**Methods:**

The presence of a second SURFIN member, SURFIN_4.1 _(PFD0100c, PFD0105c) is reported here. Bioinformatic tools were used to study the structure of the *surf*_4.1 _gene. To investigate the expression of *surf *genes PCR and real-time quantitative PCR (Rt-QPCR) were employed and Northern and Western blots were used to confirm the size of the *surf*_4.1 _gene and the SURFIN_4.1 _protein respectively. Localization of SURFIN_4.1 _was determined using immunofluorescence assays.

**Results:**

The *surf*_4.1 _gene was found present in one copy by Rt-QPCR in some parasites (3D7AH1, 3D7S8, 7G8) whereas six copies of the gene were identified in FCR3 and FCR3S1.2. *surf*_4.1 _was found transcribed in the late asexual stages of the parasite beginning ≈32 hours post invasion and throughout the schizont stages with the level of transcription peaking at late schizogony. The levels of transcript correlated with the number of gene copies in FCR3 and 3D7S8. *surf*_4.1 _was found to encode a polypeptide of ≈Mw 258 kDa (SURFIN_4.1_) present within the parasitophorous vacuole (PV), around free merozoites as merozoite-associated material, but not at the pRBC-surface. Despite multiple *surf*_4.1 _gene copies in some parasites this was not reflected in the levels of SURFIN_4.1 _polypeptide.

**Conclusion:**

SURFIN_4.1 _is a member of the SURFINs, present in the PV and on the released merozoite. The results suggest different SURFINs to be expressed at different locations in the parasite and at distinct time-points during the intra-erythrocytic cycle.

## Background

The erythrocytic life cycle of *Plasmodium falciparum *is characterized by complex interactions of parasite derived surface exposed antigens with host receptors and immune defence proteins. At the surface of the the parasitized red blood cells (pRBCs), parasite antigens provide adhesive properties and at the same time allow for limited parasite resistance to the host immune response by virtue of great sequence variability. In case of the well-characterized surface antigen *Plasmodium falciparum *erythrocyte membrane protein 1 (PfEMP1), a multi-adhesive variable ligand of 200–400 kDa, this is achieved by clonal exchange, "switching" of the mutually expressed PfEMP1 encoding *var *gene. A unique set of ≈60 *var *genes is present in each parasite genome [1-3]. With the release of merozoites from the host cell, a number of additional parasite proteins are exposed to the immune system, which, in response to the host immune pressure, show polymorphisms or are differentially expressed [4].

The merozoite, which is the invasive form of *P. falciparum*, invades erythrocytes in a cascade of events in which multiple receptor-ligand interactions facilitate parasite attachment, reorientation, junction formation and entry [5-9]. The machinery employed in this process is complex and involves proteins at the merozoite surface and in specialized secretory organelles at the merozoite apex [10]. Triggered by the initial parasite attachment to the host cell, micronemes and/or rhoptries release their contents. The Duffy binding-like (DBL) family (EBA-175 and its paralogues EBA-140 (BAEBL) and EBA-181 (JESEBL) [11,12]) in micronemes and the reticulocyte binding like (RBL) family (PfRh1, PfRh2a, PfRh2b, PfRh3 and PfRh4 [13-16]) in the neck of rhoptries have been shown to be relevant for merozoite infectivity [4]. In *P. falciparum*, several copies of Pf *Rh1 *gene were reported in FCR3, W2mef, and FCB1 (13, 3 and 4 respectively) when compared to 3D7. Thus, apart from the different protein antigens expressed on the surface of the merozoite, the complexity of the malaria parasite is increasing by the introduction of gene duplications and deletions [17-19].

SURFINs are a small family of large (200–300 kDa) proteins, which are composed of domains structurally related to PvSTP1 of *Plasmodium vivax *but also other exported and surface exposed parasite proteins [20], including PfEMP1, the *P. vivax *VIR family and *Plasmodium knowlesi *variant surface antigen (SICA*var*). In accordance with this structural relationship, SURFIN_4.2 _was found co-transported with PfEMP1 to the pRBC-surface. The protein is also observed on the merozoite as merozoite-associated material (MAM) at the apical end of the released merozoites. In the 3D7 parasite SURFINs are encoded by 10 surface-associated interspersed (*surf*) genes, which are located close or within the subtelomeric region of five chromosomes. Frequent recombination events in this area are responsible for the considerable variability of proteins encoded in this region, also observed in SURFINs. N-terminally, SURFINs contain a cysteine rich domain (CRD) defined by six conserved cysteine residues, five of which are found positionally conserved in PvSTP1. The CRD precedes a region of high size and sequence variability, followed by a putative transmembrane (TM) region. In contrast to the variable N-terminal region, the C-terminal domain of SURFINs is relatively conserved and characterized by tryptophane rich domains (WRD) of approximately 150 amino acid residues, separated by less conserved sequences [20]. The WRDs establish a structural link to several *Plasmodium *surface proteins, i.e. PfEMP1, *Pk*SICA*var *and also the giant erythrocyte membrane protein 332 (Pf332), of which recent data also suggests surface exposure [21].

The objectives of the study were to analyze the *surf*_4.1 _gene structure to determine whether it is a pseudo gene, as previously annotated, or a complete functional gene. The transcription pattern of the *surf*_4.1 _gene and localization of SURFIN_4.1 _on the protein level were also investigated.

## Methods

### Parasites and cultures

Parasites 3D7AH1, 3D7S8, FCR3, FCR3S1.2, 7G8 and TM180 were maintained in continuous culture according to standard procedures [22,23].

### Analysis of *surf *gene presence and sequence heterogeneity in various parasite lines

Based on the 3D7 sequences of the annotated SURFINs, two sets of forward and reverse primers (Primer set 1 and Primer set 2), were designed for each *surf *gene. Some of the primers were designed to cross introns while others were within the open reading frames (ORFs) (Additional Files 1 and 2). Genomic DNA was extracted from trophozoite stages of 3D7S8, NF54, 7G8, DD2, FCR3, FCR3S1.2, TM284, TM180, R29, UAM25, UKS03, and UKS05 using the DNeasy Blood and Tissue kit according to the manufacturer's instructions (Qiagen, USA). Using two sets of independently designed *surf *gene specific primers, DNA was PCR amplified from each parasite. The following were the PCR conditions 35 cycles of 94°C for 30 sec, 55°C for 30 sec, and 68°C 60 sec.

### *surf*_4.1 _transcript analysis

Highly synchronized 3D7S8 and FCR3 parasites were harvested post invasion (p.i.) at early ring (10 h), late ring (20 h), early trophozoite (30 h), and schizont (40 h) parasite stages. The parasites were resuspended in pre-warmed TRIzol (Invitrogen, USA) (37°C) and incubated at 37°C for 5 minutes. Total RNA was extracted using the manufacturer's instructions (Invitrogen, USA) and DNAse treated (Ambion, USA). The RNA (100 ng) was reverse transcribed (RT+) using MuLV reverse transcriptase enzyme and random hexamers (Applied Biosystems, USA) according to manufacturer's instructions. For each RT+ experiment, one RT- reaction (with reverse transcriptase omitted) was performed to ensure that there was no DNA contamination in the RNA.

*surf*_4.1 _is currently present as PFD0100c in GeneDB, and PlasmboDB v5.4 but it was previously indicated as a truncated gene in the PlasmoDB v4.3 malaria database and was then suggestively comprised two distinct genes PFD0105c and PFD0100c. Primers (*surf*C_4.1_F/R and Sintra_4.1 _F/R) were consequently designed on opposite sides of the intergenic region of the two previously annotated genes (yielding amplification products of 1160 bp and 450 bp respectively, see Additional File 2). cDNA from ring stages, trophozoite stages and schizont stages was amplified using *surf*C_4.1_F and *surf*C_4.1_R. The RT-PCR cycling conditions were as follows: 35 cycles of 94°C for 30 sec, 55°C for 30 sec, and 68°C 60 sec. The cDNA from the different parasite stages was also amplified using primers specific to the *surf*_4.1 _gene sections, these included 5CS-1F/R, 5CS-2F/R, *surf*C_4.1_F/R, S_4.1_F/R, 1C-S1F/R, 1C-S2F/R and 1C-S3F/R (Additional File 2).

### Identification of the PFD0105c intron location

PCR was conducted using primers *surf*C_4.1_F/R (Additional File 2) that were designed to cross the intergenic region and at the same time also read through the intron of the first part of the *surf*_4.1 _gene (PFD0105c). The primers were used to amplify cDNA and gDNA from FCR3S1.2, 3D7S8, and NF54. The cDNA and gDNA PCR products were cloned into TOPOII vector (Invitrogen USA) and prepared for sequencing using the BigDye v3.1 kit (Applied Biosystems, USA) according to manufacturer's instructions.

### Northern blot analysis

A fragment of *surf*_4.1_, *surf*_4.1_-C2 was PCR amplified using GWS_4.1_F2, 5'- atcgagctcGTACTGAACCAAAACATCAT-3' and GWS_4.1_R2, 5'-gatctcgagTGAATTAACGCTTCTTCTAGT-3' primers from parasite DNA and cloned into TOPOII vector. After sequence confirmation, labelling was performed using vector specific M13Reverse, GWSF2 and T6/T7 DIG labelling kit (Roche, Germany) according to manufacturer's instructions. Total RNA from early rings (10 h), late rings (20 h) early trophozoite (30 h), and schizonts (40 h) were subjected to agarose gel electrophoresis and transferred to a nitrocellulose membrane (0.45 μm) (BIORAD, USA). The subsequent methodology was performed as described previously [20].

### Sera and specific antibodies

A fragment of *surf *_4.1 _(from the non-cytoplasmic part of the protein), SURFIN_4.1_-C1 was PCR amplified from 3D7S8 using primers 5'-atcgagctcTTGGATAATACAGGTGAT-3' and 5'-gatctcgagTTCCTTATGATGTTTTGGT-3'. The amplified DNA was cloned into TOPOII vector and sequenced. The SURFIN_4.1_-C1 fragment was subsequently cloned into the PQE Trisystem -His strep 2 expression vector (Qiagen, USA) which adds a stretch of 6 histidine residues to the C terminus and 8 streptavidin residues at the N-terminus. The fusion protein was purified on His-Trap columns (Amersham Biotec, Sweden) charged with Nickel ions (1 M, NiCl_2_, SIGMA, USA) according to manufacturer's instructions. The purified fusion protein was used to immunize rabbits (500 μg/injection) and rats (100 μg/injection) in Freund's complete adjuvant (SIGMA, USA) and boosted three times at one-month intervals with the same protein concentration in Freund's incomplete adjuvant. The animals were bled 10 days after the last boost.

### Western blot analysis

Late stage FCR3 and 3D7S8 pRBCs from ≈32 h onwards were lysed in SDS sample buffer. Extracts (5 × 10^7 ^cells/lane) were separated on a 4–15% gradient SDS- PAGE (BIORAD). Proteins were transferred onto nitrocellulose membranes (0.45 μm) (BIORAD) and transiently stained with 0.1% Ponceau S in acetic acid. The membranes were blocked with 5% milk-PBS-0.05%Tween20 overnight (O/N) at 4°C, and then incubated with rabbit-anti- SURFIN_4.1_-C1 purified IgG, polyclonal antibodies (1: 200) in blocking buffer O/N at 4°C. Goat-anti-rabbit Ig coupled to alkaline phosphatase (1:10,000, Amersham Biotech, Sweden) was used as secondary antibody and the protein was visualized using 5-bromo-4-chloro-3-indolyl phosphate/nitroblue tetrazolium (BCIP/NBT) (SIGMA) in water.

### Real-time quantitative PCR analysis of *surf*_4.1 _copy numbers and transcription levels

Copy numbers of *surf*_4.1 _were determined for 7G8, 3D7AH1, FCR3 and their daughter clones 3D7S8 and FCR3S1.2. Genomic DNA (gDNA) was prepared using Easy-DNA™ Kit (Invitrogen) as previously described [17]. In addition, transcriptional levels were analysed for 3D7S8 and FCR3. Parasites were kept tightly synchronized using 5 % (w/v) sorbitol, and were harvested at 4 h post invasion intervals for two consecutive parasite generations. RNA from each time point was isolated using the RNeasy^® ^Mini Kit according to the manufacturer's instructions and contaminating gDNA was removed using the RNase-Free DNase Set (Qiagen). Total RNA was reversely transcribed with SuperScript III RNase H reverse transcriptase (Invitrogen), with random hexamers and oligo(dT)_12–18 _(300 ng/ml and 25 ng/ml respectively; Invitrogen) at 50°C for two hours. For each cDNA synthesis reaction, a control reaction without reverse transcriptase (RT-) was performed with identical amounts of template. For real-time quantitative PCR (Rt-QPCR) determination of copy numbers as well as to monitor relative transcription of *surf*_4.1 _to the endogenous control *seryl-tRNA synthetase*, the primers *surf*_4.1 _were employed:

5'-TTTGAAGCTCCTGGTCAAGGA-3', *surf*_4.1_-3': TTGTTTGTGCAAGTGTTTTGAAAG, PF07_0073-5': TATCATCTCAACAGGTAT CTACATCTCCTA and PF07_0073-3': TTTGAGAGTTACATGTGGTATCATCTTTT. These primers as well as the complete Rt-QPCR amplified product were designed to conserved regions in the parasite lines studied. Amplification efficiencies of the primer pairs were tested on dilution series of both gDNA and cDNA from all parasites, and were proven sufficiently close (ranging from 94.4 to 99.2%) to obviate the need for a correction factor. Amplification reactions for both relative copy numbers (gDNA) and relative transcription (cDNA) were done in quadruplicate in 20 μl, containing Power SYBR Green master mix (Applied Biosystems), 300 nM of each forward and reverse primer and 2 ng of template. Forty five cycles (95°C for 15 sec and 60°C for 1 min) were performed in an ABI sequence detector 7500 (Applied Biosystems). Relative copy numbers were analysed according to the ΔΔCt method and plotted using SigmaPlot 9.0 (Systat Software Inc.). Transcription levels were achieved by dividing log-transformed Ct values ($2^{\text{-}\bar{x}\text{ Ct}surf4.1}/2^{\text{-}\bar{x}\text{ Ct}seryl\text{-}tRNA\;synthetase}$) for each strain and time point (for details see Additional File 6). The standard deviation of the quotient was calculated according to the User Bulletin 2, Applied Biosystems. Results were visualized as log_2 _transformed values plotted using SigmaPlot 9.0 (Systat Software Inc.).

### Indirect immunofluorescence on air-dried monolayers and localization of SURFIN_4.1_

For immunofluorescence assays (IFA), monolayers of pRBCs were prepared as previously described [24]. Monolayers were incubated with rabbit anti-SURFIN_4.1_-C1 antibody (1:400) or pre-immune serum for 30 min at room temperature and subsequently with a secondary anti-rabbit Alexa 488 (1:100; Jackson, USA) for 30 min. The cells were then incubated with propidium iodide (1:100) for nuclear staining for 30 min. Preparations were washed three times with PBS between each antibody preparation.

In the co-localization assay with EBA175, monolayers were incubated with rabbit anti-SURFIN_4.1_-C1(1:100) for 30 min and thereafter with anti-rabbit -TRITC (1:100, Jackson, USA) for another 30 min. Monolayers were then probed with rat anti-EBA175 (MR-2) (1:400; ATCC/MR4) and after three washes with PBS probed with secondary anti-rat Alexa 488 antibody.

When SURFIN_4.1 _was evaluated for its co-localization with SURFIN_4.2 _and MSP1, monolayers were probed with rat anti-SURFIN_4.1_-C1 (1:100) for 30 min, washed three times and incubated with secondary chicken-anti-rat Alexa 488 for 30 min. The monolayers were then incubated with rabbit anti-SURFIN_4.2 _(1:200) or rabbit anti-MSP1 FVO (1:400, ATCC/MR4) for 30 min and then probed with secondary goat-anti-rabbit Ig- TRITC (1:100; Jackson, USA) for 30 min. HOESCHT and/or DAPI (Jackson, USA) were used for nuclear staining. All incubations were carried out at room temperature in a humid chamber. Slides were analysed with a Nikon Optiphot 2 UV microscope.

### Agglutination assays

To analyse the ability of the immune anti-SURFIN_4.1_-C1serum to agglutinate pRBCs, synchronized parasite cultures of 3D7S8 and FCR3 were incubated with serum as described by Barragan *et al *[25]. The samples were then analysed under the microscope for presence of clumps or agglutinates. Acridine orange was used for staining of the parasite.

## Results

**Figure 1:**
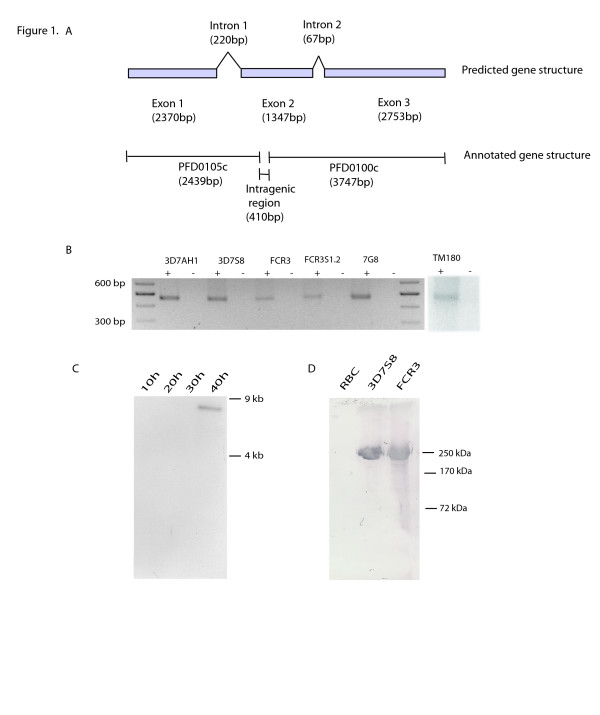
***surf*_4.1 _from gene to protein**. A). The new *surf*_4.1 _gene structure, showing three exons separated by two introns. Previously the gene was annotated as two genes (PFD0100c and PFD0105c). Using the new structure, with the two genes combined, the protein structure of SURFIN_4.1 _is estimated to be a 258.3 kDa protein. B). PCR with Sintra_4.1_F/R primers amplified the intergenic region separating the two genes which make up *surf*_4.1 _(PFD0105c and PFD0100c). PCR on cDNA shows presence of transcript in 3D7AH1, 3D7S8, FCR3, FCR3S1.2, 7G8, and TM180 parasite strains. + and - indicate cDNA treated with reverse transcriptase (+) and one not treated with reverse transcriptase (-) respectively. C). Northern blot on 3D7S8, 10 hrs, 20 hrs, 30 hrs and 40 hrs RNA with *surf*_4.1 _specific probe. A band of an approximately 7 kb is observed from RNA extracted at 40 h post invasion. The *surf*_4.1 _RNA has an open reading frame with a spliced size of 6471 bp. D). Immunoblot on whole parasite cultures of 3D7S8 and FCR3 from 40 hrs^+ ^probed with rabbit anti-SURFIN_4.1 _purified IgG show a band above 250 kDa confirming that SURFIN_4.1 _is a full protein encoded by a complete *surf*_4.1 _gene.*surf *_4.1 _gene encodes a protein estimated at 258.3 kDa.

The data presented here shows a revised gene structure of *surf*_4.1_, which combines two previously identified open reading frames (PFD0100c and PFD0105c) (plasmodb.org v4.3 and Figure 1A). This was obtained by bioinformatic analysis and confirmed by PCR, which shows that *surf*_4.1 _is one complete gene with three exons separated by two introns (Figure 1B). In PlasmoDB, *surf*_4.1 _gene was previously annotated as two individual open reading frames, PFD0100c and PFD0105c separated by a 410 bp intergenic region (hereby referred to as intergenic region). However, RT-PCR amplification using primers designed to amplify the fragment surrounding this intergenic region, showed a single band from cDNA indicating that the annotation was incorrect and that these two predicted open reading frames compose a single open reading frame. This led to the recent re-annotation of the gene in PlasmoDB v5.4. In this study the *surf*_4.1 _transcript was shown to be a single open reading frame in 3D7S8, FCR3, FCR3S1.2, 3D7AH1, 7G8 and TM180 parasite lines (Figure 1B).

### *surf*_4.1 _and other *surf *genes exhibit sequence heterogeneity in laboratory parasites and field isolates

The presence of *surf*_4.1 _in laboratory strains and clinical isolates was investigated. PCR on gDNA with *surf*_4.1 _specific primers resulted in specific amplification in all parasite lines (Additional File 3). The remaining *surf *genes were also amplified, some with only one primer set while others were amplified using two primer sets. These two separate primer sets were used to account for any sequence differences and to ensure that as many *surf *genes were amplified from the different parasites. Genomic DNA sequences from 3D7, HB3, D10, DD2 and 7G8 from *surf*4.1 (1–2320 bp.) showed sequence polymorphisms within the parasite isolates (Additional File 4).

### *surf*_4.1 _exists in different copy numbers in different parasite strains

**Figure 2:**
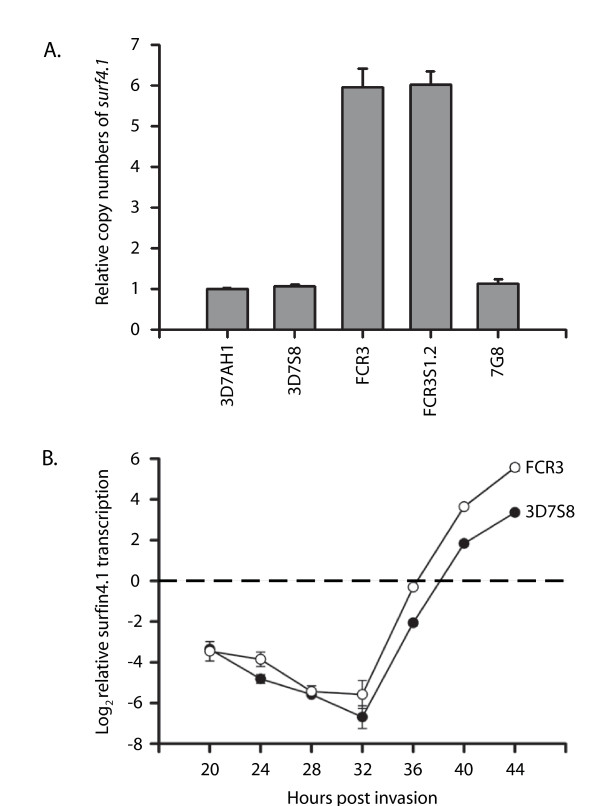
***surf*_4.1 _copy numbers and transcription pattern**. A). Rt-QPCR analysis of *surf*_4.1 _copy numbers in 3D7AH1, 3D7S8, FCR3, FCR3S1.2 and 7G8 parasite strains revealed FCR3 and FCR3S1.2 to have six copies relative to the other strains. The relative copy number determination was conducted using *seryl-tRNA synthetase *as the endogenous control gene. B). Rt-QPCR reveals that *surf*_4.1 _transcription is initiated at ≈32 h post invasion and peaks during late schizogony. FCR3 shows ≈5-fold higher level of transcription compared to 3D7S8 at 44 h post invasion, corresponding well to the gene copy number abundance in respective genomes. Results are visualized as log_2 _transformed values.

Six copies of *surf*_4.1 _gene were observed in FCR3 and its daughter clone FCR3S1.2 and one copy in each of 3D7AH1, 3D7S8, and 7G8 (Figure 2A and Additional File 5). Achieved copy numbers confirm previous findings where microarray and fluorescent *in situ *hybridizations were used for quantification [18].

### *surf *genes are differentially transcribed dependent on developmental stage and strain

To understand the transcription patterns of the *surf *genes in the 3D7S8 parasite clone, RT-PCR was performed with primers specifically targeting individual sequences of all 10 *surf *genes (Additional File 1). Different *surf *genes were found expressed at different times during the erythrocytic cycle (Table 1). *surf*_1.3_, *surf*_4.2 _and *surf*_8.3 _were expressed throughout the cycle, from the early rings to the schizonts, while other *surf *genes were either not detected (*surf*_1.2_, *surf*_8.1_, *surf *_8.2 _and *surf*_13.1_) or restricted to later trophozoites and/or schizont development (*surf*_1.1_, *surf*_4.1 _and *surf*_14.1_) (Table 1). *surf*_4.1 _was found prominently transcribed in late stages in the two parasite lines 3D7S8 and FCR3, with an apparent onset of transcription at ≈32 h and peaking in late schizonts (Figure 2B and Additional File 6). An obvious difference in transcript levels was observed between these two parasite lines, corresponding to the difference in copy numbers of the *surf*_4.1 _gene.

**Table 1:** Developmental expression of surf genes as seen by RT-PCR using primer set 1. *surf* genes exhibit a differential expression pattern during the different stages of the parasite cycle.

*surf *Gene		**Time Post Invasion (hrs)**				SURFIN Grouping^§^
			
	PlasmoDB ID	**10**	**20**	**30**	**40**	
***surf*_1.1_**	PFA0625w	-	-	+	+	**A**
***surf*_1.2_**	PFA0650w	-	-	-	-	***Truncated***
***surf*_1.3_**	PFA0725w	+	+	+	+	**B**
***surf*_4.1_**	PFD0105c, PFD0100c	-	-	+	+	**A**
***surf*_4.2_**	PFD1160w	+	+	+	+	**A**
***surf*_8.1_**	MAL8P1.1	-	-	-	-	**B**
***surf*_8.2_**	PF08_0002	-	-	-	-	**A**
***surf*_8.3_**	MAL8P1.162	+	+	+	+	**B**
***surf*_13.1_**	PF13_0074, PF13_0075	-	-	-	-	**Truncated**
***surf*_14.1_**	PF14_0747	-	-	+	-	**B**

The genes that are expressed are indicated by "+" while those that are not expressed are indicated by "-". *surf*_4.1_ is expressed in the late stages of the cycle from ≈30 hrs all the way until the dividing merozoites. Three *surf* genes (*surf*_1.3_, *surf*_4.2_ and *surf*_8.3_) are expressed throughout the life cycle while others are expressed at a particular stage in the life cycle.

+ = Gene amplified.

- = Gene not amplified.

^§^SURFIN grouping is based on the number of exons [20] as well as protein alignments which have resulted in *surf*_4.1 _to be in group A even though it has 3 exons. The transmembrane domain in the group A paralogs is present in the first exon and in the second exon in group B paralogs

The transcription pattern of *surf*_4.1 _was confirmed with Northern blot experiments using specific probes. *surf*_4.1 _mRNA of approximately 9 kB was detected in schizonts > 40 h post infection (Figure 1C).

### Identification of SURFIN_4.1 _by immunoblotting

Immunoblot analysis was performed with SDS-lysates obtained from synchronized *in vitro *cultures of 3D7S8 and FCR3. Using polyclonal rabbit-anti-SURFIN_4.1_-C1 antibodies, a band of approximately 250 kDa was detected in schizont stage parasites (36–40 h), which corresponds to the predicted SURFIN_4.1 _protein mass of 258 kDa (Figure 1D). Similar intensities were observed in both FCR3 and 3D7S8. Achieved data is consistent with the RT-PCR analysis suggesting PFD0100c and PFD0105c to form a single open reading frame.

### SURFIN_4.1 _is localized to the parasitophorous vacuole (PV)

**Figure 3:**
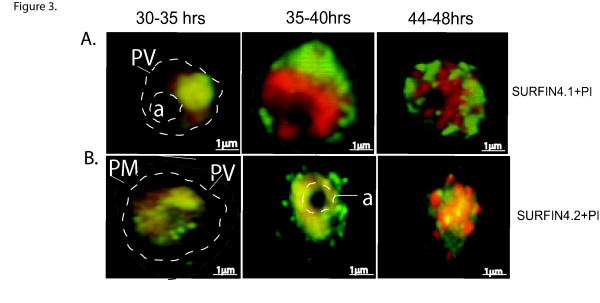
**Localization of SURFIN_4.1 _by immunofluorescence staining on air-dried monolayers from 3D7S8 parasite strain**. Air dried monolayers probed with rabbit anti-SURFIN_4.1 _on 3D7S8 pRBC. Propidium iodide (red) was used to stain the parasite nucleus and SURFIN_4.1 _and SURFIN_4.2 _proteins were stained green using anti-rabbit Alexa 488. SURFIN_4.1 _localizes within the parasitophorous vacuole (PV) and is observed from approximately 30 hrs post invasion. SURFIN_4.1 _was observed as a green dot above the food vacuole (**a**), at 30–35 hrs parasite stages. The protein was spread around the parasitophorous vacuole (PV) at 35–40 hrs parasite stages and in the mature schizont (44–48 hrs) SURFIN_4.1_was observed between the dividing merozoites. During the trophozoite and early schizont stages SURFIN_4.2 _shows a similar pattern of staining as SURFIN_4.1_.

**Figure 4:**
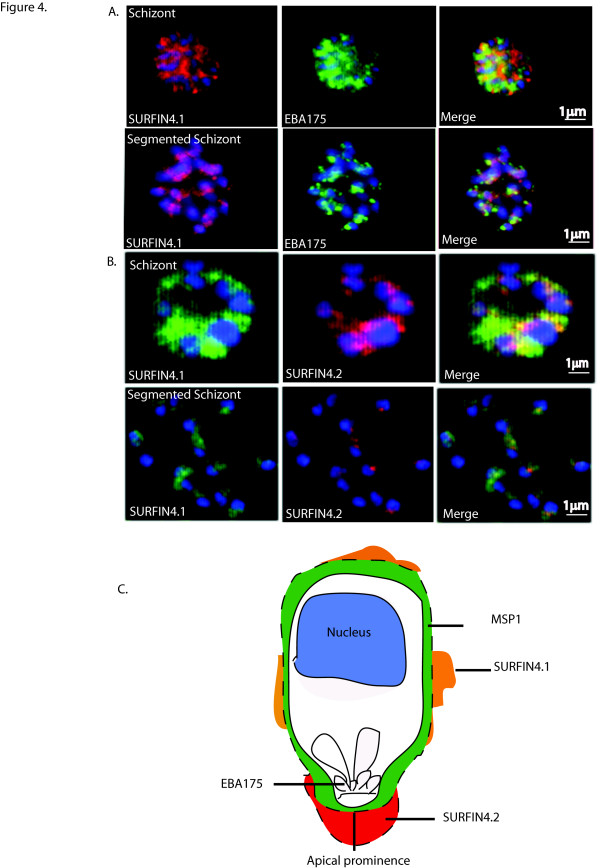
**Localization of SURFIN_4.1 _in respect to other merozoite associated proteins by immunofluorescence staining**. A). Co-localization study between rat anti-SURFIN_4.1 _and rabbit anti-EBA175 was carried out on 3D7S8 air dried monolayers. EBA175 is a micronemal protein hence localizes at the merozoite apex. The parasite nucleus was stained in blue using Hoescht. In the intact schizont SURFIN_4.1 _and EBA175 partially co-localize as shown in the merge of the two photos. B). Co-localization between SURFIN_4.1 _(green) and SURFIN_4.2 _(red) is observed in the intact schizont as indicated by the yellow colour in the merged photos. In the ruptured schizont on the other hand, SURFIN_4.1 _(green) is spread around the merozoites (blue) while SURFIN_4.2 _(red) is observed as a distinct dot on the merozoite (blue). The merge of the two colocalization patterns shows that SURFIN_4.1 _(green) colocalizes with SURFIN_4.2 _(red) even though SURFIN_4.1 _(red) is more spread out around the merozoite compared to SURFIN_4.2 _which is present at the apex of the merozoite (red). C). A graphical outline of a merozoite showing locations of known merozoite proteins, MSP1 and EBA175 in relation to SURFIN_4.1 _and SURFIN_4.2 _is depicted here. MSP1 is shown in green surrounding the merozoite, EBA175 is shown in the micronemes, SURFIN_4.1_in orange shown as patches of MAM around the merozoite and SURFIN_4.1 _in red as MAM at the apical end of the merozoite.

To study the localization of SURFIN_4.1_, IFA was carried out on 3D7S8 and FCR3 air-dried monolayers using purified rabbit-anti-SURFIN_4.1_-C1 IgG. SURFIN_4.1 _was found expressed during the mature stages of the parasite (30 h and onwards, Figure 3). There was no recognition of SURFIN_4.1 _during the early ring stages (0–16 h) or in trophozoite stages (16–24 h). In the late trophozoite stages (25–30 h) SURFIN_4.1 _was observed close to the food vacuole (FV) in the PV as a distinct spot, which later spread out within the PV in a dotty pattern (Figure 4) in both 3D7S8 and FCR3.

During late schizont stage, SURFIN_4.1 _was seen as merozoite associated material (MAM) around the newly formed merozoites in intact schizonts. After schizont rupture SURFIN_4.1 _localized around each individual merozoite (Figure 3). When a SURFIN_4.2 _antibody was used on the same parasite stages, the same pattern of staining was achieved for both trophozoite and early schizont stages. However in the ruptured schizont, SURFIN_4.2 _antibody showed a distinct staining of the merozoite apex while the pattern observed with SURFIN_4.1 _antibody was not apical but rather spread around the merozoite (Figure 3).

Co-localization experiments were carried out between SURFIN_4.1 _and the micronemal protein EBA175, and SURFIN_4.2_. In the intact schizont, SURFIN_4.1 _appears to co-localize in part with EBA175, but the proteins differentiate in the ruptured schizont. SURFIN_4.1 _is spread around the pre-released merozoites, while EBA175 localizes at the apical end of the merozoite (Figure 4A). SURFIN_4.1 _is also co-localized with SURFIN_4.2 _in the intact schizont but again the two proteins differentiate in the ruptured (segmented) schizont as SURFIN_4.1 _is spread around the pre-released merozoites while SURFIN_4.2 _is also seen on the merozoites but with apical staining (Figure 4A–B).

### SURFIN_4.1 _seems not to be exposed on the infected erythrocyte surface

Agglutination assay on pRBCs and RBC binding assay on uninfected RBCs with rabbit-anti-SURFIN_4.1_-C1 whole serum showed no agglutinates in either 3D7S8 or FCR3 (30 h and 40 h cultures).

## Discussion

SURFINs of *P. falciparum*, encoded by the *surf *multi gene family, compose a new family of surface proteins. Not much is known about the protein family and its function [20]. This paper reports on the identification of SURFIN_4.1 _a member of the SURFINs present in the parasitophorous vacuole (PV) and in part at the level of the merozoite. SURFIN_4.1 _is encoded by the *surf*_4.1 _gene, which, as is SURFIN_4.2 _(the only member of the family thoroughly studied), located on chromosome 4 of *P. falciparum*.

In order to investigate the presence of *surf *genes in both laboratory strains and clinical isolates of *P. falciparum*, PCR was carried out on all the 10 *surf *genes using two different primer sets for each gene. Two independent primer sets were used in order to reduce bias in gene amplification, which may arise with the use of only one primer set due to sequence variation (Additional File 3). The outcome showed that all the genes were amplified in either laboratory adapted or clinical isolates, but not every gene was amplified in every parasite (Additional File 3).

In the same manner the *surf*_4.1 _gene was also found amplified in all parasite lines, but for isolate R29 (Additional File 3). Single nucleotide polymorphisms (SNPs) were observed in laboratory strains (HB3, DD2, D10, 7G8, IT, RO33, K1, FCR3, and FCC-2), some clinical isolates from Uganda (UAM25, UKS03 and UKS05) and GHANA 1 from Ghana (Additional File 4). The sequence data on *surf*_4.1 _from HB3, DD2, D10, 7G8 (Additional File 4) further confirms the existence of sequence heterogeneity of the *surf*_4.1 _gene.

A detailed analysis of the gene revealed that *surf*_4.1 _is one complete gene comprised of three exons with one continuous open reading frame (ORF) (Figure 1A) [20]. Differences in length of the first intron were observed between FCR3 and 3D7S8 parasite strains with FCR3 having a longer intron. It is not established whether the difference in intron length and or presence of SNPs could contribute to the *surf*_4.1 _gene maintaining a complete open reading frame in certain *P. falciparum *strains. The gene encodes a 258 kDa protein, SURFIN_4.1_, which is of a slightly smaller molecular weight than the previously characterised SURFIN_4.2 _(286.4 kDa) (Figure 1D).

With the knowledge that *surf*_4.1 _exists as one complete gene, copy number polymorphisms were analysed in different parasite isolates. FCR3 and its daughter clone FCR3S1.2 (cloned by micromanipulation) were found to have six copies of the gene, in concordance with copy number estimates performed previously using microarrays [18]. The increase in *surf*_4.1 _gene copy numbers correlated with a five-fold increase in RNA transcription observed in FCR3 compared to the 3D7S8 parasite strain. However, the increase in copy number of the gene was not reflected on level of protein, rather that the protein was present a similar levels in the two parasites. This might be due to sequence differences in the protein in FCR3, resulting in poorer recognition by the antibodies raised using a protein construct from 3D7S8 And another possibility may be that there is a certain level of translational repression as has been previously described in *P. falciparum *[26].

The temporal transcriptional profile of *surf*_4.1 _was analysed in the 3D7S8 and FCR3 parasite strains. *surf*_4.1 _is transcribed during the late stages from ≈32 h post invasion peaking at the late schizont stages (44–48 hrs). The difference in transcription patterns between *surf*_4.1 _(late trophs and schizonts) and *surf*_4.2 _(transcribed from early rings to the schizont stages) may suggest differences in function of the proteins encoded by the respective genes. The transcription profile of *surf*_4.1 _correlated well with the protein expression, observed in both FCR3 and 3D7S8 parasite lysates (Figure 3). No detectable protein for SURFIN_4.1 _was observed in early parasite stages.

In the trophozoite stage, SURFIN_4.2 _is present in the PV as is SURFIN_4.1_, which appears during the late trophozoite or early schizont stages and is observed in segmented schizonts carrying newly formed merozoites. SURFIN_4.1 _is also observed as amorphous material (MAM) around the merozoites, which could suggest that it plays a role in the merozoite invasion process. It is interesting to note that SURFIN_4.1 _partially co-localises with both SURFIN_4.2 _and EBA175 in the intact schizont but the proteins differentiate in the segmented schizonts. Both SURFIN_4.2 _and EBA175 are observed at the apical end of merozoites in the segmented schizont while SURFIN_4.1 _is spread around the merozoite (Figure 4A–B).

SURFIN_4.1 _seems not to be exposed on the pRBC surface as shown by failure of the anti-SURFIN_4.1_-C1 serum to agglutinate IE in FCR3 and 3D7S8. This observation is in line with the IFA on air-dried monolayer results, which indicates that SURFIN_4.1 _remains within the PV and is not present in the pRBC cytoplasm or at the surface of IE. Immunofluorescence assay on live cells showed no reactivity with SURFIN_4.1_-C1 serum. Still both SURFINs have PEXEL-like motifs: SURFIN_4.1 _has a PEXEL-like motif (RNVFE) located at aa 25–29 while in SURFIN_4.2 _the PEXEL motif is RKIFE though at the same aa position. The motifs differ but whether this difference could affect the differential transport of SURFINs to the IE is to be elucidated.

The function of SURFIN_4.1 _is not yet known, but other members of the SURFINs have been associated with putative functions such as SURFIN_4.2_, which might be involved in erythrocyte invasion. PFA0650w (*surf*_1.2_) has been suggested to be involved in surface adhesion [27] and PFD0100c (part of *surf*_4.1 _gene) has been shown to share Myb-protein domains and GO annotations with PFI1480w and PFL0815w, which have putative functions regarding transcriptional regulation [28].

Antigenic variation has been reported in some multi-gene families including the *var *gene family [29-31]. The *surf *multi-gene family shows differential expression of its genes (Table 1). Some of the genes are expressed simultaneously during the entire blood stage parasite cycle; some are expressed throughout the life cycle while one third is expressed during the later stages of the cycle. It is not yet established whether differential expression of the *surf *genes could suggest different functions.

## Conclusion

In summary, *surf*_4.1 _is one complete, and not truncated, gene in *P. falciparum *parasites, with gene-copy number polymorphisms existing amongst different clones and strains. The *surf*_4.1 _gene encodes a SURFIN_4.1 _protein of Mw ≈ 258 kDa present in the PV and associated with the released merozoite.

## Websites

Websites Genedb: 

Plasmodb: 

Applied Biosystems: 

## Abbreviations

SURFIN: surface associated interspersed protein family; CRD: cysteine rich domain; WRD: tryptophane rich domain; PV: parasitophorous vacuole; PBS: Phosphate buffered saline, pRBC: parasitized red blood cells; RT-PCR: reverse transcript-PCR; Rt-QPCR: Realtime-quantitative PCR; IFA: immunofluorescence assay; EM: erythrocyte membrane; FV: food vacuole; MAM: merozoite associated material.

## Authors' contributions

FM carried out the experiments except the real-time quantitative PCR which was done by UR. OK designed primer set 2 and did part of the sequencing. GW, FK and MW conceived and designed the study and participated in the drafting and coordination of the manuscript.

## Supplementary Material

Additional file 1 Click here for file

Additional file 2 Click here for file

Additional file 3 Click here for file

Additional file 4 Click here for file

Additional file 5 Click here for file

Additional file 6 Click here for file
