# Supplementary material for: SURFIN4.1, a schizont-merozoite associated protein in the SURFIN family of Plasmodium falciparum
Source: Malar J. 2008 Jul 1;7:116. doi: 10.1186/1475-2875-7-116 (PMC2515329; doi:10.1186/1475-2875-7-116)
Supplement: Additional file 1 [file 1475-2875-7-116-S1.doc]

# Additional File 1. *surf* Gene Specific Primers (Primerset 1, Primerset 2)

| **Gene** | **PlasmoDB ID** | **5’ Primer Sequence (Primerset 1) 3’** | **5’ Primer Sequence (Primerset 2) 3’** |
| --- | --- | --- | --- |
| ***surf*_1.1_F** | PFA0625w | GCTTGGATTTTAGGTGC | AATGAAAGTAGCGAATACCCTGTAG |
| ***surf*_1.1_R** | PFA0625w | CTTCCCAAACATACATATAG | TTTTCCATTCTTCTTTTCTAATATCTTC |
| ***surf*_1.2_F** | PFA0650w | CCATCTTGCTCAACCGATGAATGACAT | CCCATTTTATCAATAAAACAAATCAAAG |
| ***surf*_1.2_R** | PFA0650w | CTGCTACTAACACTACTGTAACTTAAATAA | GACACTTTTTATATGTTTATTCATCATAC |
| ***surf*_1.3_F** | PFA0725w | GACATAGTTCTCCAGGTTTA | TCATGCGGAATCTGGAATGTTTGG |
| ***surf*_1.3_R** | PFA0725w | CAGCAGAAGGAACCGC | TTCTATTTTTGTTTCTCTTATGCCTTG |
| ***surf*_4.2_F** | PFD1160w | CTTCCCCTTTACAAATGAATGCTC | AATATTATCAATGTTAGGTTTGTC |
| ***surf*_4.2_R** | PFD1160w | AACATCAACACCTCTACGCCGC | AAAATATATAATCATCTTGATCATC |
| ***surf*_8.1_F** | MAL8P1.1 | CCATTCTTCATTTTTCCATTTTTTTTTTTTG | GTCAATAATACACTTGTTCTAGAAGG |
| ***surf*_8.1_R** | MAL8P1.1 | CAGCAAGATTGGATTAGGAGAG | TCACTCATCACTTCCAAGTCGTC |
| ***surf*_8.3_F** | MAL8P1.162 | GACATAGTTCTCCAGGTTTA | TCATGCGGAATCTGGAATGTTTAG |
| ***surf*_8.3_R** | MAL8P1.162 | CGTGAAGAAATGAGAGAAAAAG | TTTCTAATTCTTTTTCTCTCATTTCTTC |
| ***surf*_13.1_F** | PF13_0074, PF13_0075 | GACCATGTACGCTTGAAGAATC | ATTACAACAAGATATGTTCCAATTACC |
| ***surf*_13.1_R** | PF13_0074, PF13_0075 | CCGTGTAGGGAACCT | TCTTTTATTATATTATCTTCCTCTTGTG |
| ***surf*_14.1_F** | PF14_0747 | GACTATTCTGGTTTTCCCTC | TGGATACATTAACATCTGAAAATTCTC |
| ***surf*_14.1_R** | PF14_0747 | AGTATGTCCCCGTAGCTTTAGCAGT | TTCTATTTTTGTTTCTCTTATGCCTTG |

**Additional file 1: *surf* gene specific PCR primers used for amplification**

Two sets of specific primers were designed from the 3D7 *P.falciparum* reference strain for each *surf* gene. These primers were used to amplify each of the *surf* genes from gDNA and cDNA.
