# Supplementary material for: SURFIN4.1, a schizont-merozoite associated protein in the SURFIN family of Plasmodium falciparum
Source: Malar J. 2008 Jul 1;7:116. doi: 10.1186/1475-2875-7-116 (PMC2515329; doi:10.1186/1475-2875-7-116)
Supplement: Additional file 2 [file 1475-2875-7-116-S2.doc]

**Additional File 2: *surf*_4.1_ (PFD0100c) Primers**

| **Section** | **5’ Primer Sequence 3’** |
| --- | --- |
| **^*^ *surf*C_4.1_F** | CCTCACAATTTTTCCAGTCGCA |
| **^*^ *surf*C_4.1_R** | AGTATGTCCCCGTAGCTTTAGCAGT |
| **S_4.1_F** | ATGTTGGAAAAAAAGTATAAATGTGAA |
| **S_4.1_R** | TCCATATATATTTCTATAACGGTTTTC |
| **5C-S1F** | ATGCATTTTGTAGTTGAA |
| **5C-S1R** | AATATGTTTAGGTTTCAGCC |
| **5C-S2F** | GGCTGAAACCTAAACATATT |
| **5C-S2R** | ACTGCTAAAGCTACGGGGACATACT |
| **1C S1F** | TGCGACTGGAAAAATTGTGAGG |
| **1C-S1R** | GTTTTTTCTTTCCCCTTCTTTC |
| **1C-S2F** | GGAAAGAAGGGGAAAGAAAAAAC |
| **1C-S2R** | CCTATCCTTTTATTCATTTCC |
| **1C-S3F** | GGAAATGAATAAAAGGATAGG |
| 1C-S3R | CACAAGAATTATTAAATATTTCG |
| **§Sintra_4.1_F** | GAAGAAGGTATGATGTGA |
| **§Sintra_4.1_R** | CTTCACATTTATACTTTTTTTC |

*Primers crossing the intergenic region between PFD0100c and PFD0105c with *surf*C_4.1_F in PFD0100c and *surf*C_4.1_R in PFD0105c open reading frames (ORFs).

§Short primers designed crossing the intergenic region between PFD0100c and PFD0105c with the Sintra_4.1_F in PFD0105c ORF and Sintra_4.1_ R in PFD0100c ORF.

**Additional file 2: *surf*_4.1_ specific primers**

*surf*_4.1_ specific primers designed from the 3D7 *P. falciparum* reference strain. The primers were designed for the different sections of the gene and were used in amplifying both gDNA, cDNA.

Note that two primer sets were designed to amplify the intergenic region, *surf*C_4.1_F/R (1160bp product) and Sintra_4.1_F/R (450bp product).
