# Supplementary material for: SURFIN4.1, a schizont-merozoite associated protein in the SURFIN family of Plasmodium falciparum
Source: Malar J. 2008 Jul 1;7:116. doi: 10.1186/1475-2875-7-116 (PMC2515329; doi:10.1186/1475-2875-7-116)
Supplement: Additional file 3 [file 1475-2875-7-116-S3.doc]

***Additional File 3. Genomic amplification of surf genes in different P.falciparum isolates by PCR using surf gene specific primers (Primerset 1 or Primerset 2; see footnote and Additional File 1).***

| ***surf* gene** | | | | | | | | | | |
| --- | --- | --- | --- | --- | --- | --- | --- | --- | --- | --- |
| **Parasite** | **Origin** | ***surf*_1.1_** | ***surf*_1.2_** | ***surf*_1.3_** | ***surf*_4.1_** | ***surf*_4.2_** | ***surf*_8.1_** | ***surf*_8.3_** | ***surf*_13.1_** | ***surf*_14.1_** |
| **3D7S8** [1] | Stockholm clone of 3D7 | + | + | + | + | + | + | + | + | + |
| **NF54** [2] | Netherlands | + | + | + | + | + | + | + | + | + |
| **7G8** [3] | Brazil | - / + | - / + | + / - | + / - | + / - | - / - | + | - | + |
| **DD2*** [4] | Indochina | + | - | - | + | + | - | + | + | - |
| **FCR3** [5] | The Gambia | + | - / + | + / - | + / - | + | + | - | - / + | - |
| **FCR3S1.2** [6] | Stockholm clone of FCR3 | + | - / + | + / - | + / - | + | + | - | - | + / - |
| **TM284** [7] | Thailand | + / - | - / + | + / - | + | - / + | + / - | - | - | + / - |
| **TM180** [7] | Thailand | - | - | + | + | - / + | + | - / + | - | + / - |
| **R29*** [8] | Brazil | - | - | + | - | + | - | + | - | + |
| **UAM25*** [9] | Apac in Uganda | + | - | + | + | + | + | + | - | + |
| **UKS03** [10] | Kampala in Uganda | + / - | + | + / - | + / - | + / - | + | + | - / + | + |
| **UKS05** [10] | Kampala in Uganda | - / + | + | + / - | + / - | + / - | + | + | - | - |

*Only one primer set used (primer set 1).

+ = present (with both primer sets except in *).

- = not amplified (with both primer sets except in *).

+/ - = amplified by primer set 1 and not amplified by primer set 2 (for further details as to the primer sets see Additional file 1).

-/ += amplified by primer set 2 and not by primer set 1 (for further details as to the primer sets see Additional file 1).

.

**Additional file 3: Genomic amplification of *surf* genes in different *P.falciparum* isolates by PCR using *surf* gene specific primers (Primer set 1 or Primer set 2; see footnote).**

Different *surf* genes (gDNA) were amplified by PCR using two primer sets, set 1 and set 2, both designed from 3D7 reference strain. The genes that were amplified were indicated by “+” while those that are not amplified are indicated by “-”. Those genes, which were amplified by only one primer set but not the other are indicated as “-/+ or +/- “. All the 10 *surf* genes were at least amplified from different laboratory or wild *P.falciparum* strains as outlined in the table. The two primer sets complemented each other making it possible to amplify most of the *surf* genes in the different parasite isolates.

1. Winter G, Chen Q, Flick K, Kremsner P, Fernandez V, Wahlgren M: **The 3D7var5.2 (var COMMON) ty+e var gene family is commonly expressed in non-placental Plasmodium falciparum malaria**. *Mol Biochem Parasitol* 2003, **127**(2):179-191.

2. Delemarre BJ, van der Kaay HJ: **Tropical malaria contracted the -natural way in the Netherlands**. *Ned Tijdschr Geneeskd* 1979, **123**(46):1981-1982.

3. Graves PM, Carter R, Burkot TR, Rener J, Kaushal DC, Williams JL: **Effects of transmission-blocking monoclonal antibodies on different isolates of Plasmodium falciparum**. *Infect Immun* 1985, **48**:611-616.

4. Guinet F, Dvorak JA, Fujioka H, Keister DB, Muratova O, Kaslow DC, Aikawa M, Vaidya AB, Wellems TE: **A Developmental defect in Plasmodium falciparum male gametogenesis.**.*J Cell Bio*1996,**135**:269-278.¨’

5. Jensen JB, Trager W: **Plasmodium falciparum in culture: establishment of additional strains**. *Am J Trop Med Hyg* 1978, **27**:743-746.

6. Fernandez V, Treutiger CJ, Nash GB, Wahlgren M. **Multiple adhesive phenotypes linked to rosetting binding of erythrocytes in Plasmodium falciparum malaria**. *Infect Immun* 1998, **66**:2969-2975.

7. Treutiger CJ, Scholander C, Carlsson J, McAdam KP, Raynes JG, Falksveden L, Wahlgren M: **Roleaux-forming serum proteins are involved in the rosetting of Plasmodium falciparum infected erythrocytes**. *Exp Parasitol* 1999, **93**:215-224.

8. Roberts DJ, Craig AG, Berendt AR, Pinches R, Nash G, Marsh K, Newbold CI. **Rapid switching to multiple antigenic and adhesive phenotypes in malaria.** *Nature* 1992, **357:**689-692.
