# Supplementary material for: SURFIN4.1, a schizont-merozoite associated protein in the SURFIN family of Plasmodium falciparum
Source: Malar J. 2008 Jul 1;7:116. doi: 10.1186/1475-2875-7-116 (PMC2515329; doi:10.1186/1475-2875-7-116)
Supplement: Additional file 5 [file 1475-2875-7-116-S5.doc]

## **Additional File 5. Relative gene copy numbers of *surf_4.1_* in various parasite strains and clones.**

| Strain | Gene | $\bar{x}$_Ct gene_^*^ | ΔCt | ΔΔCt | Relative Quantity | CI^‡^ |
| --- | --- | --- | --- | --- | --- | --- |
| 3D7AH1 | *seryl-tRNA synthetase* | 21.20 | 0.00 | 0.00 | 1 | 0.02 |
| “ | *surf_4.1_* | 21.08 | -0.12 | 0.00 | 1 | 0.03 |
| 3D7S8 | *seryl-tRNA synthetase* | 20.01 | 0.00 | 0.00 | 1 | 0.06 |
| “ | *surf_4.1_* | 19.79 | -0.22 | -0.10 | 1.07 | 0.05 |
| FCR3 | *seryl-tRNA synthetase* | 19.89 | 0.00 | 0.00 | 1 | 0.11 |
| “ | *surf_4.1_* | 17.19 | -2.70 | -2.58 | 5.96 | 0.45 |
| FCR3S1.2 | *seryl-tRNA synthetase* | 20.23 | 0.00 | 0.00 | 1 | 0.03 |
| “ | *surf_4.1_* | 17.52 | -2.71 | -2.59 | 6.01 | 0.34 |
| 7G8 | *seryl-tRNA synthetase* | 21.57 | 0.00 | 0.00 | 1 | 0.13 |
| “ | *surf_4.1_* | 21.25 | -0.32 | -0.20 | 1.14 | 0.11 |

^*^ Averaged Ct-values from quadruplicate amplification reactions.

^‡^ Confidence interval using 95% confidence level.

**Additional file 5: Relative gene copy numbers of *surf_4.1_* in various parasite strains and clones.**

This table displays detailed information of the relative copy number estimations of *surf*_4.1_ in 3D7AH1, 3D7S8, FCR3, FCR3S1.2 and 7G8 performed with Rt-QPCR.
