# Supplementary material for: SURFIN4.1, a schizont-merozoite associated protein in the SURFIN family of Plasmodium falciparum
Source: Malar J. 2008 Jul 1;7:116. doi: 10.1186/1475-2875-7-116 (PMC2515329; doi:10.1186/1475-2875-7-116)
Supplement: Additional file 6 [file 1475-2875-7-116-S6.doc]

## **Additional File 6. Differential transcription of *surf_4.1_* in parasite lines FCR3 and 3D7S8.**

| Strain | Time p.i.  (h) | Gene | $\bar{x}$_Ct gene_ | 2^-Ctgene^ | Normalized gene value^*^ | Log_2_ Normalized gene value | Log_2_ SD of quotient ^‡^ |
| --- | --- | --- | --- | --- | --- | --- | --- |
| FCR3 | 20 | *seryl-tRNA synthetase* | 24.94 | 3.11^-8^ | 1 | 0 | 0.07 |
| “ | 20 | *surf_4.1_* | 28.48 | 2.83^-9^ | 0.091 | -3.46 | 0.48 |
| “ | 24 | *seryl-tRNA synthetase* | 25.39 | 2.28^-8^ | 1 | 0 | 0.12 |
| “ | 24 | *surf_4.1_* | 29.29 | 1.57^-9^ | 0.069 | -3.85 | 0.36 |
| “ | 28 | *seryl-tRNA synthetase* | 24.75 | 3.57^-8^ | 1 | 0 | 0.16 |
| “ | 28 | *surf_4.1_* | 30.20 | 8.25^-10^ | 0.023 | -5.43 | 0.28 |
| “ | 32 | *seryl-tRNA synthetase* | 24.92 | 3.15^-8^ | 1 | 0 | 0.08 |
| “ | 32 | *surf_4.1_* | 30.62 | 6.58^-10^ | 0.021 | -5.58 | 0.69 |
| “ | 36 | *seryl-tRNA synthetase* | 24.99 | 3.01^-8^ | 1 | 0 | 0.12 |
| “ | 36 | *surf_4.1_* | 25.30 | 2.42^-8^ | 0.806 | -0.31 | 0.10 |
| ” | 40 | *seryl-tRNA synthetase* | 25.00 | 2.99^-8^ | 1 | 0 | 0.12 |
| ” | 40 | *surf_4.1_* | 21.36 | 3.72^-7^ | 12.448 | 3.64 | 0.10 |
| ” | 44 | *seryl-tRNA synthetase* | 24.31 | 4.83^-8^ | 1 | 0 | 0.12 |
| ” | 44 | *surf_4.1_* | 18.75 | 2.28^-6^ | 47.319 | 5.56 | 0.17 |
| 3D7S8 | 20 | *seryl-tRNA synthetase* | 24.64 | 3.84^-8^ | 1 | 0 | 0.11 |
| ” | 20 | *surf_4.1_* | 28.00 | 3.74^-9^ | 0.097 | -3.36 | 0.13 |
| ” | 24 | *seryl-tRNA synthetase* | 22.10 | 2.23^-7^ | 1 | 0 | 0.04 |
| ” | 24 | *surf_4.1_* | 26.94 | 7.90^-9^ | 0.035 | -4.82 | 0.22 |
| ” | 28 | *seryl-tRNA synthetase* | 23.80 | 6.86^-8^ | 1 | 0 | 0.03 |
| ” | 28 | *surf_4.1_* | 29.38 | 1.43^-9^ | 0.021 | -5.58 | 0.10 |
| ” | 32 | *seryl-tRNA synthetase* | 25.05 | 2.87^-8^ | 1 | 0 | 0.06 |
| ” | 32 | *surf_4.1_* | 31.83 | 2.78^-10^ | 0.010 | -6.69 | 0.55 |
| ” | 36 | *seryl-tRNA synthetase* | 25.19 | 2.62^-8^ | 1 | 0 | 0.06 |
| ” | 36 | *surf_4.1_* | 27.26 | 6.27^-9^ | 0.239 | -2.06 | 0.14 |
| ” | 40 | *seryl-tRNA synthetase* | 25.33 | 2.37^-8^ | 1 | 0 | 0.07 |
| ” | 40 | *surf_4.1_* | 23.50 | 8.43^-8^ | 3.561 | 1.83 | 0.08 |
| ” | 44 | *seryl-tRNA synthetase* | 25.58 | 2.00^-8^ | 1 | 0 | 0.07 |
| ” | 44 | *surf_4.1_* | 22.22 | 2.05^-7^ | 10.261 | 3.36 | 0.06 |

^*^ Normalized gene values were calculated according to (2^-Ctgene^)/( 2^-Ct^ *^seryl-tRNA synthetase^*)

^‡^ Standard deviation of quotient computed according to the “Standard deviation calculation using the standard curve method” in Applied Biosystems User Bulletin #2 (www.appliedbiosystems.com)

**Additional file 6: Differential transcription of *surf_4.1_* in parasite lines FCR3 and 3D7S8.**

This table displays detailed information of the transcriptional pattern and levels of *surf*_4.1_ in 3D7S8 and FCR3achieved using Rt-QPCR.
