# Supplementary material for: SURFIN4.1, a schizont-merozoite associated protein in the SURFIN family of Plasmodium falciparum
Source: Malar J. 2008 Jul 1;7:116. doi: 10.1186/1475-2875-7-116 (PMC2515329; doi:10.1186/1475-2875-7-116)
Supplement: Supplementary file 6 [file 1475-2875-7-116-S4.doc]

```
          10        20        30        40        50        60        70        80        90
3D7  CATTTTGTAGTTGAATTGGATAATACAGGTGATGATTTTAATGATAAGGCTATATCCATTGAAAGATTTAGAAATGTTTTTGAGGTTTATGTTGAAG
7G8  --------AGTTGAATTGGATAATACAGGTGATGATTTTAATGATAAGGCTATATCCATTGAAAGATTTAGAAATGTTTTTGAGGTTTATGTTGAAG
D10  CATTTTGTAGTTGAATTGGATAATACAGGTGATGATTTTAATGATAAGGCTATATCCACTGAAAGATTTAGAAATGTTTTTGAGGTTTATGTTGAAG
Dd2  CATTTTGTAGTTGAATTGGATAATACAGGTGATGATTTTAATGATAAGGCTATATCCACTGAAAGATTTAGAAATGTTTTTGAGGTTTATGTTGAAG
HB3  CATTTTGTAGTTGCATTGGATAATACAGGTGATGATTTTAATGATAAGGCTATATCCACTGAAAGATTTAGAAATGTTTTTGAGGTTTATGTTGAAG

         110       120       130       140       150       160       170       180       190
3D7  AAATAGATGAATTAGATACAAAACCATCAGAAATACTTAATAAAGAATGTCGACATTTTAATTATTTTATTGATGATATGAAAGATGAGTTTCTCAC
7G8  AAATAGATGAATTAGATACAAAACCATCAGAAATACTTAATAAAGAATGTCGACATTTTAATTATTTTATTGATGATATGAAAGATGAGTTTCTCAC
D10  AAATAGATGAATTAGATACAAAACCATCAGAAATACTTAATAAAGAATGTCGACATTTTAATTATTTTATTGATGATATGAAAGATGAGTTTCTCAC
Dd2  AAATAGATGAATTAGATACAAAACCATCAGAAATACTTAATAAAGAATGTCGACATTTTAATTATTTTATTGATGATATGAAAGATGAGTTTCTCAC
HB3  AAATAGATGAATTAGATACAAAACCATCAGAAATACTTAATAAAGAATGTCGACATTTTAATTATTTTATTGATGATATGAAAGATGAGTTTCTCAA

         210       220       230       240       250       260       270       280       290
3D7  TTCATTAATACGTCTCCCCAAAAAATTAAGAAAGCAATTATGGGAAAGTGAAGTTGATGAAAATTTACCAAATTTAATGGCTACAACTACACATAAT
7G8  TTCATTAATACGTCTCCCCAAAAAATTAAGAAAGCAATTATGGGAAAGTGAAGTTGATGAAAATTTACCAAATTTAATGGCTACAACTACACATAAT
D10  TTCATTAATACGTCTCCCTAAAGAATTAAGAAAGCAATTATGGGAAAATGAAGTTGATAAAAACTTACCAAATTTAATGGCTACAACTACACATAAT
Dd2  TTCATTAATACGTCTCCCTAAAAAATTAAGAAAGCAATTATGGGAAAGTGAAGTTGATGAAAACTTACCAAATTTAATGGCTACAACTACACATAAT
HB3  TTCATTAATACGTCTCTCTAAAGAATTAAGAAAGCAATTATGGGAAAGTGAAGTTGATGAAAAATTACCAAATTTAATGGCTACAACTACACATAAT

         310       320       330       340       350       360       370       380       390
3D7  TGTCTAAGAACAGAACATAATTATGATAAAAAGTATAGGGACGTCATTAAAATATTAGAAGATTATTGTGAAGATAGGGCTACTAAATTGAGGGATT
7G8  TGTCTAAGAACAGAACATAATTATGATAAAAAGTATAGGGACGTCATTAAAATATTAGAAGATTATTGTGAAGATAGGGCTACTAAATTGAGGGATT
D10  TGTCTAAGAACAGAACATAATTATGATAAAAAGTATAGGGACGTCATTAAAATATTAGAAGATTATTGTGAAGATAGGGCTACTAAATTGAGGGATT
Dd2  TGTCTAAGAACAGAACATAATTATGATAAAAAGTATAGGGACGTCATTAAAATATTAGAAGATTATTGTGAAGATAGGGCTACTAAATTGAGGGATT
HB3  TGTCTAAGAACAGAACATAATTATGATAAAAAGTATAGGGACGTCATTAAAATATTAGAAGATTATTGTGAAGATAGGGCTACTAAATTGAGGGATT

         410       420       430       440       450       460       470       480       490
3D7  AAGCCATAAAATATGCAGAACAGGATTGTATTAATTTTAATACCTGGGTAAGTAGTTGGGACTATGAAATAAAAAGGCAAATGAATAAATTAGATAT
7G8  AAGCCATAAAATATGCAGAACAGGATTGTATTAATTTTAATACCTGGGTAAGTAGTTGGGACTATGAAATAAAAAGGCAAATGGATAAATTAGATAT
D10  AAGCCATAAAATATGCAGAACAGGATTGTATTAATTTTAATACCTGGGTAAGTAGTTGGGACTATGAAATAAAAAGGCAAATGAATAAATTAGATAT
Dd2  AAGCCATAAAATATGCAGAACAGGATTGTATTAATTTTAATACCTGGGTAAGTAGTTGGGACTATGAAATAAAAAGGCAAATGAATAAATTAGATAT
HB3  AAGCCATAAAATATGCAGAACAGGATTGTATTAATTTTAATACCTGGGTAAGTAGTTGGGACTATGAAATAAAAAGGCAAATGGATAAATTAGATAT

         510       520       530       540       550       560       570       580       590
3D7  AAAAATAAAACAATATTTGGAAAAGTCAAAGTTCAAATGTGATATAAATGATTTAGATAATTTAGATACATTTTTTTCCCATGTAAATCCAAAAGAT
7G8  AAAAATAAAACAATATTTGGAAAAGTCAAAGTTCAAATGTGATATAAATGATTTAGATAATTTAGATACATTTTTTTCCCATGTAAATCCAAAAGAT
D10  AAAAATAAAACAATATTTGGAAAAGTCAAAGTTCAAATGTGATATAAATGATTTAGATAATTTAGATACATTTTTTTCCCATGTAAATTCAAAAGAT
Dd2  AAAAATAAAACAATATTTGGAAAAGTCAAAGTTCAAATGTGATATAAATGATTTAGATAATTTAGATACATTTTTTTCCCATGTAAATCCAAAAGAT
HB3  AAAAATAAAACAATATTTGGAAAAGTCAAAGTTCAAATGTGATATAAATGTTTTAGATAATTTAGATACATTTTTTTCCCATGTAAATTCAAAAGAT

         610       620       630       640       650       660       670       680       690
3D7  AAGGGAAGTGAAGAGGATCCAAGAAATCCTCAGGAAGAAGAGCACAAAAGAGATGAATATCAAGTTTTAAATAGTACAGTTGAAGAAGGTGATGAAT
7G8  AAGGGAAGTGAAGAGGATCCAAGAAATCCTCAGGAAGAAGAGCACAAAAGAGATGAATATCAAGTTTTAAATAGTACAGTTGAAGAAGGTGATGAAT
D10  AAGGGAAGTGAAGAGGATCCAAGAAATACTCAGGAAGAAGAGCACAAAAGAGATGAATATCAAGTTTTAAATAGTACAGTTGAAGAAGGTGATGAAT
Dd2  AAGGGAAGTGAAGAGGATCCAAGAAATCCTCAGGAAGAAGAGCACAAAAGAGATGAATATCAAGTTTTAAATAGTACAGTTGAAGAAGGTGATGAAT
HB3  AAGGGAAGTGAAGAGGATCCAAGAAATACTCAGGAAGAAGAGCACAAAAGAGATGAATATCAAGTTTTAAATAGTACAGTTGAAGAAGGTGATGAAT

         710       720       730       740       750       760       770       780       790
3D7  TTGATAGAATAAATATCCTTTCTACAGAAACAGGCGAAGCGACACCAAATATTCAAGAAGAACCTTTAAGGGTTACTCCTCATGGAGGGGATAATCA
7G8  CTGATAGAAAAAATATGCCTTCTATAGAAACAGGCGAAGAGACACCAAATATTCAAGAAGAACCTTTAAGGGTTACTCCTCATGGAGGGGATGATCA
D10  TTGATAGAATAAATATCCTTTCTACAGAAACAGGCGAAGCGACACCAAATATTCAAGAAGAACCTTTAAGGGTTACTCCTCATGGAGGGGATAATCA
Dd2  CTGATAGAAAAAATATCCCTTCTATAGAAACAGGCGAAGAGACACCAAATGTTCAAGAAGAACCTTTAAGGGTTACTCCTCATGGAGGGGATGATCA
HB3  CTGATAGAATAAATATCCCTTCTATAGAAACAGGCGAAGAGACACCAAATGTTCAAGAAGAACCTTTAAGGGTTACTCCTCATGGAGGGGATGATCA

         810       820       830       840       850       860       870       880       890
3D7  TAAAACACCTCCTACAATTGTAAAGACTGAAGTAGGTAATGATAATCGAGAGAATGTAGTTTCTACTGCTGGTAAAAGATTAAATAGAGGTAAACCA
7G8  TAAAACACCTCCTACAATTGTAAAGACTGAAGTAGGTAATGATAATCGAGAGAATGTAGTTTCTACTGCTGGTAAAAGATTAAATAAAGGTAAACTA
D10  TAAAACACCTCCTACAATTGTAAAGACTGAAGTAGGTAATGATAATCGAGAGAATGTAGTTTCTACTGCTGGTAAAAGATTAAATAGAGGTAAAGCA
Dd2  TAAAACACCTCCTACAATTGTAAAGACTGAAGTAGGTAATGATAATCGAGAGAATGTAGTTTCTACTGCTGGTAAAAGATTAAATAGAGGTAAAGCA
HB3  TAAAACACCTCCTACAATTGTAAAGACTGAAGTAGGTAATGATAATCGAGAGAATGTAGTTTCTACTGCTGGTAAAAGATTAAATAAAGGTAAACTA
```

```
                       10        20        30        40        50        60        70        80
               ....|....|....|....|....|....|....|....|....|....|....|....|....|....|....|....|....|...
PFD0100c_7G8   --XVELDNTGDDFNDKAISIERFRNVFEVYVEDKIDELDTKPSEILNKECRHFNYFIDDMKDEFLTSSLIRLPKKLRKQLWESEVDEN
D10            HFVVELDNTGDDFNDKAISTERFRNVFEVYVEDKIDELDTKPSEILNKECRHFNYFIDDMKDEFLTSSLIRLPKELRKQLWENEVDKN
Dd2            HFVVELDNTGDDFNDKAISTERFRNVFEVYVEDKIDELDTKPSEILNKECRHFNYFIDDMKDEFLTSSLIRLPKKLRKQLWESEVDEN
HB3            HFVVALDNTGDDFNDKAISTERFRNVFEVYVEDKIDELDTKPSEILNKECRHFNYFIDDMKDEFLKSSLIRLSKELRKQLWESEVDEK
3D7            HFVVELDNTGDDFNDKAISIERFRNVFEVYVEDKIDELDTKPSEILNKECRHFNYFIDDMKDEFLTSSLIRLPKKLRKQLWESEVDEN

                      110       120       130       140       150       160       170       180
               ....|....|....|....|....|....|....|....|....|....|....|....|....|....|....|....|....|...
PFD0100c_7G8   CLRTEHNYDKKYRDVIKILEDYCEDRATKLRDLKAIKYAEQDCINFNTWVSSWDYEIKRQMDKLDISKIKQYLEKSKFKCDINDLDNL
D10            CLRTEHNYDKKYRDVIKILEDYCEDRATKLRDLKAIKYAEQDCINFNTWVSSWDYEIKRQMNKLDISKIKQYLEKSKFKCDINDLDNL
Dd2            CLRTEHNYDKKYRDVIKILEDYCEDRATKLRDLKAIKYAEQDCINFNTWVSSWDYEIKRQMNKLDISKIKQYLEKSKFKCDINDLDNL
HB3            CLRTEHNYDKKYRDVIKILEDYCEDRATKLRDLKAIKYAEQDCINFNTWVSSWDYEIKRQMDKLDISKIKQYLEKSKFKCDINVLDNL
3D7            CLRTEHNYDKKYRDVIKILEDYCEDRATKLRDLKAIKYAEQDCINFNTWVSSWDYEIKRQMNKLDISKIKQYLEKSKFKCDINDLDNL

                      210       220       230       240       250       260       270       280
               ....|....|....|....|....|....|....|....|....|....|....|....|....|....|....|....|....|...
PFD0100c_7G8   KGSEEDPRNPQEEEHKRDEYQVLNSTVEEGDEYADRKNMPSIETGEETPNIQEEPLRVTPHGGDDHVKTPPTIVKTEVGNDNRENVVS
D10            KGSEEDPRNTQEEEHKRDEYQVLNSTVEEGDEYVDRINILSTETGEATPNIQEEPLRVTPHGGDNHVKTPPTIVKTEVGNDNRENVVS
Dd2            KGSEEDPRNPQEEEHKRDEYQVLNSTVEEGDEYADRKNIPSIETGEETPNVQEEPLRVTPHGGDDHVKTPPTIVKTEVGNDNRENVVS
HB3            KGSEEDPRNTQEEEHKRDEYQVLNSTVEEGDEYADRINIPSIETGEETPNVQEEPLRVTPHGGDDHVKTPPTIVKTEVGNDNRENVVS
3D7            KGSEEDPRNPQEEEHKRDEYQVLNSTVEEGDEYVDRINILSTETGEATPNIQEEPLRVTPHGGDNHVKTPPTIVKTEVGNDNRENVVS

                      310       320       330       340       350       360       370       380
               ....|....|....|....|....|....|....|....|....|....|....|....|....|....|....|....|....|...
PFD0100c_7G8   KEGGQHTTKFKTPKVSASVGGKKSKNLRGNTEPKHQKEKTSYDRTRKTSEQINKYAHPNNKYTIFQCTDLDCKWLKPKNIKYTYEGHD
D10            KDGVPHTTKFKTPKVRAPAEGKKSKNARDSTEPKHQKEKRPYDRIKKTREQINKYAHPDNKYTIFQCTDLDCKWLKPKHIKYPYEGYD
Dd2            KDGVPHTTKFKTPKVRAPAEGKKSKNARDSTEPKHHKEKGKYDRIKKTSEQINKYAHPNNKYTIFQCTDLDCKWLKPKHIKYPYEGYD
HB3            KEGGQHTTKFKTPKVSASVGGKKSKNLRGNTEPKHQKEKTSYDRTRKTSEQINKYAHPNNKYTIFQCTDLDCKWLKPKNIKYTYEGYD
3D7            KDGVPHTSRFKTPKVSAPAGGKKSKNARDSTEPKHHKEKGKYDRIKKTSEQINKYAHPNNKYTIFQCTDLDCKWLKPKHIKYPYEGYD

                      410       420       430       440       450       460       470       480
               ....|....|....|....|....|....|....|....|....|....|....|....|....|....|....|....|....|...
PFD0100c_7G8   GVYEDYNKYCSGDECALGNAVEGQFDEIGSEGNLKRKKGKGKGRKKDHXXSVGKSLPNGEGTHSYMRHGDNKEETKSQSHSVSEISAE
D10            GDYEDHHTYCSGDECAFGNAVGGQFDESRSQGKQRRKKGKGKGRKKGHNKLEGKSSPNVEGTHSYITNGGNIEETKSQSYSVTDNYVE
Dd2            GDYEDHHTYCSGDECAFGNAVEGQFDESRSQGKQRRKKGKGKGRKKGHXXSVGKSLPNGEGTHSYITNGGNREETKSQSYSVTDNYVE
HB3            GVYEDYNKYCSGDECALGNAVEGQFDEIGSEGNLKRKKGKGKGRKKDHXXSVGKSLPNGEGTHSYMRHGDNKEETKSQSHSVTDHDVE
3D7            GDYEDHHTYCSGDECAFGNAVGGQFDESRSQGKQRRKKGKGKGRKKGHNKLEGKSSPNVEGTHSYITNGGNIEETKSQSYSVTDNYVE

                      510       520       530       540       550       560       570       580
               ....|....|....|....|....|....|....|....|....|....|....|....|....|....|....|....|....|...
PFD0100c_7G8   CIDPTKQTFVIEGNTVRSMEIGDVPKMETVTSIDLPKAINEVPSSVGAHLEDKEQLLDFTIQPVIYPSDENDAFVHEVAVTYDPNLLP
D10            CIDLIKDVFIIEGGRVIRTSINDVPQMEAITNKYMPGSIYENPSSGSSHIEDKGQLKDFTIETVKYTSDGLNGFDHEVAVT-DPKLLP
Dd2            CIDLIKDEFIIEGGRFIRTSINDVPQMEAITNKYMPGSIYENPSSGSSHIEDKGQLKDFTIETVKYTSDGLNGFDHEVAVT-DPKLLP
HB3            CIDPTKQIFVIEGDHVRSISINNIPKMEAVTSIDSPKGINEVPSSVGARVEDKGQLLDFTIEPVIYQSDENNGFVHEEPVT-DPKLLP
3D7            CIDLIKDEFIIEGGRFIRTSINDVPQMEAITNKYMPGSIYENPSSGSSHIEDKGQLKDFTIETVKYTSDGLNGFDHEEPVT-DPKLLH

                      610       620       630       640       650       660       670       680
               ....|....|....|....|....|....|....|....|....|....|....|....|....|....|....|....|....|...
PFD0100c_7G8   TALPDYNEIALHGNPIPHQGFSPRYSSNSITSIEGTSNNVSTIESLTSIIGNFVDAIRNSVISKRSTNSALEFSESTTRPESSYSLPQ
D10            ESLPDHNEIVFYGSPIPHRRFARSYPLNTTTTLEGTSKKPSTIESLTSLFENFFDSIKSSSRTRRSVNQDLELSESAAMTSSTSPLRQ
Dd2            ESLPHHNDIALNGSPIPHRRFARSYPLNTTTTLEGTSQKPSAMETIMSLFSNFFDSIKSSSITRRSVKHDLELSESTTSPTNSYSLPQ
HB3            ESLPHHNDIALHGNPIQYQGFSPRYSSNSITSIEGTSNNVSTIESLTSIIGNFVDAIKSSVISKRSINSALEFSESTTRPESSYSLPQ
3D7            ESLPHHNDIALHGSPIPRRRFSRSYPLNTTTTLEGTSQKPSAMETIMSLFRNIFDSIKSSSITRRSVNSALEFSESTTRPGNSFSLPQ

                      710       720       730       740       750       760       770
               ....|....|....|....|....|....|....|....|....|....|....|....|....|....|....|
PFD0100c_7G8   VMGMLGNGNSYTSLAQKSQEVSSSSPVSRSSSFNHNSQTINRILKAQVDGTYVVSG-HKVNVPKGIEHYIKSVPV
D10            VMGMLGQVNSYTSLGQTKQEVASSSPGSPSHSFTLRSKTMHPILKRQFEGTYVVSG-HKANVPKGIEHYIKYVPV
Dd2            VMRMLGQKNSFKTLAQTKQEVSSSSSESRSSSFSHNSQIMPTILKRPINGNYVVSGGHKTNVPKGIEHYIKYVPV
HB3            VMRMLGNGNSYTSLAQKSQEVSSSSPVSRSSSFNHNSQTINRILKAQVDGTYVVSG-HKVNVPKGIEHYIKSVPV
3D7            VMRMLGQKNSFKTLAQTKQEVSSSSSESRSSSFNNNSQIIPHILKTQVDGTYVVSG-HKANVPKGIEHYIKYVPV
```
